# Simulated auditory nerve response evoked by lateral-wall and peri-modiolar electrode arrays inserted in scala tympani or scala vestibuli

**DOI:** 10.3389/fnins.2025.1639092

**Published:** 2025-12-17

**Authors:** Cornelia Wenger, Andreas Fellner, Frank Rattay

**Affiliations:** Institute for Analysis and Scientific Computing, Vienna University of Technology, Vienna, Austria

**Keywords:** cochlear implant, scala vestibuli, auditory nerve fibers, finite element model, multi-compartment model, scala tympani ossification

## Abstract

**Introduction:**

Cochlear implants (CIs) are neuroprosthetic devices designed to restore hearing in individuals with severe to profound hearing loss. Clinically, CI electrode arrays differ by location, either peri-modiolar (pm) positioned near the cochlear axis, or lateral-wall (lw), placed closer to the peripheral terminals of auditory nerve fibers (ANFs). Standard insertion is into the scala tympani (ST), though the scala vestibuli (SV) is considered for cases of severe ST ossification.

**Methods:**

This computational study investigates the neural responses of both healthy and degenerated ANFs to monopolar stimulation from electrode arrays at different locations. Using a 3D finite element model of the human cochlea, we evaluated threshold excitations for short monophasic pulses across 25 traced ANF pathways. Four array configurations were compared (pmST, pmSV, lwST, lwSV), each with electrodes directly targeting the traced fibers.

**Results:**

Results show that excitation thresholds and stimulation specificity depend on both neural health and array position. Specificity, defined by minimizing off-target fiber stimulation, was highest for healthy ANFs with lwSV, followed by pmST, lwST, and pmSV, influenced by electrode orientation. Simulated ST ossification, modeled as decreased conductivity, generally led to reduced cathodic thresholds, suggesting that ST insertion may still be advantageous even with developing or partial ossification.

**Discussion:**

The pmST array consistently offered reliable outcomes across varying electrode configuration and neural conditions. These findings support considering SV insertion when ST is obstructed and highlight factors influencing CI performance based on array location and neural status.

## 1 Introduction

A cochlear implant (CI) is an electronic device for individuals with severe to profound hearing loss which is designed to directly stimulate their auditory nerves. A CI consists of an external part that is worn behind the ear, with a microphone, a speech processor, and a transmitter. Another part of the CI, the electrode array, is surgically placed inside the cochlea. According to the place theory (tonotopic principle) the insertion depth of every contact is linked to the local Eigenfrequency of the basilar membrane (Rattay and Lutter, 1997; Potrusil et al., 2020). The auditory perception of a CI user depends on the artificially generated spiking pattern in the afferent spiral ganglion cells, which connect the auditory sensory cells (inner hair cells) to the cochlear nucleus, the first auditory processing center in the brainstem. Each bipolar spiral ganglion cell functions as a cable composed of three segments: dendrite, soma, and axon. However, hearing deficits are often accompanied by dendrite loss (Spoendlin and Schrott, 1989) or reduced dendritic diameter (Heshmat et al., 2020). Cochlear implantation outcomes and individual auditory performance are highly variable and depend on several factors, including cognitive skills and neural status (Walia et al., 2023), CI coding strategy (Cucis et al., 2018; Wagner et al., 2024), and electrode array insertion depth (Schatzer et al., 2014; Landsberger et al., 2015). Insertion depth is measured in millimeters or by the insertion angle *α* around the cochlear axis, where α = 0° marks the array’s entry at the round window and α = 360° indicates the end of the first turn. A study on neural tonotopy in 14 unilateral CI patients (Vermeire et al., 2008) found that the observed frequency-place function did not significantly shift from the logarithmic frequency-place relationship of Greenwood’s function for the healthy human cochlea, where 20 kHz corresponds to *α* = 0° and 1 kHz to α = 360° (Greenwood, 1961). However, studies on frequency-to-place mismatch in CI users are numerous (Canfarotta et al., 2020; DeFreese et al., 2025), and challenges such as noise reduction (Carlyon and Goehring, 2021) and improving the limited perception of music (Limb and Roy, 2014; Sharp et al., 2018) and environmental sounds (Shafiro et al., 2022) remain active areas of research.

Despite these challenges, the CI is considered the most successful neuroprosthetic device, with global users exceeding one million (Zeng, 2022). Various CI designs are commercially available, differing in materials, array dimensions, number and orientation of electrodes, and insertion location (Dhanasingh and Jolly, 2017; Zeng et al., 2008). Typically, there are peri-modiolar (pm) arrays, which are placed near the cochlear axis, and lateral-wall (lw) arrays, which are closer to the peripheral terminals of the targeted auditory nerve fibers (ANFs). Clinical studies have not definitively established the superiority of one design over the other.

A retrospective review of 119 adult CI patients (Sturm et al., 2021) suggested that pm arrays may provide better speech perception within the first 6 months after CI insertion, though performance levels between pm and lw arrays equalized by 24 months. A study of 14 patients with single-sided deafness found a smaller place-pitch mismatch for pm arrays (Peters et al., 2019), whereas lw arrays were more effective for intensity discrimination (Kreft et al., 2004; Rattay and Tanzer, 2022). However, analysis of a CI database with 328 patients (Fabie et al., 2018) revealed no statistically significant differences in audiological outcomes among lw, mid-scalar, and pm arrays after adjusting for preoperative residual hearing and speech recognition.

The standard insertion site is the scala tympani (ST). However, CI candidates often have some degree of cochlear obstruction in the ST (Rinia et al., 2006), which complicates surgery or prevents full array insertion. Insertion problems may also lead to mislocated electrode contacts. For instance, a meta-analysis (Jwair et al., 2020) showed that scalar translocation and tip fold-overs, which negatively affect hearing performance, occur more frequently with pm arrays. Scalar translocation (Finley et al., 2008) refers to the electrode arrays that penetrate from the ST into the scala vestibuli (SV), damaging sensitive structures such as the Organ of Corti or the osseous spiral lamina. Tip fold-overs mainly occur in the 270° region, which hinders stimulation of ANFs in the medium and lower frequency regions. Thus, lw arrays are preferred for minimizing intracochlear trauma (Jwair et al., 2020; Mistrík et al., 2017).

As an alternative, electrode array insertion into the scala vestibuli has been clinically tested for several decades. Steenerson et al. (1990) inserted the electrode array in the SV of two patients with post-meningitis ST obstruction caused by ossification and fibrosis, and results showed outcomes comparable to those expected for an ST electrode array. Reports comparing audiological outcomes for ST and SV electrodes are contradictory. Some studies found slightly better performance with SV electrode arrays (Balkany et al., 1996; Kiefer et al., 2000; Bacciu et al., 2002; Aronson and Arauz, 2004; Lee S. et al., 2019; Trudel et al., 2018), while others reported significantly lower word recognition scores with SV electrodes (Finley et al., 2008; O'Connell et al., 2016; Shaul et al., 2018).

Computational studies on ANF excitation can provide deeper insights into the principles governing spiking patterns and may help to clarify inconsistent clinical findings. Typically, CI simulation studies use a two-step approach: first, the electrical field is calculated within a volume conductor model of the implanted cochlea, and second, the responses of selected ANFs are computed with a cable model (e.g., Rattay et al., 2001a; Kalkman et al., 2014; Hanekom and Hanekom, 2016; Nogueira et al., 2016; Bai et al., 2019; Frijns et al., 2000; Sriperumbudur et al., 2020; Potrusil et al., 2020; Croner et al., 2022; Fellner et al., 2024).

In this study, we use a three-dimensional finite element model of the human cochlea featuring a dataset of 25 traced ANFs (Potrusil et al., 2020; Fellner et al., 2024) to simulate their neural response to CI stimulation. We analyze monopolar stimulation at four electrode array locations: a pm and a lw array in each scala (pmST, pmSV, lwST, lwSV). To make them directly comparable, we created laterally aligned arrays at equivalent locations, maintaining consistent distances between active electrode contacts and target ANFs. Threshold excitation profiles are evaluated using short monophasic pulses for both healthy ANFs and two forms of neural degeneration, represented by either a reduced dendritic diameter or the absence of the dendrite. The effects of progressive ST ossification, simulated by decreased ST conductivity, are also examined, along with an analysis of how different conductivity assumptions within key regions and pulse polarity influence outcomes.

## 2 Materials and methods

To analyze ANF excitation from CI stimulation, we developed a computational pipeline using Python scripts. We used a finite element model of the human cochlea with an electrode array (implemented in COMSOL Multiphysics 6.1: https://www.comsol.com) to calculate the extracellular potential along ANF pathways. Using the MPh 1.2.3 Python interface^1^, COMSOL was controlled directly from Python, enabling automated model parameter setup, simulation execution, and results extraction. Induced ANF spiking was then simulated with a compartment model (Rattay et al., 2001b) via NEURON’s Python interface (Carnevale and Hines, 2006).

### 2.1 Volume conductor model of the human cochlea

**Figure 1:**
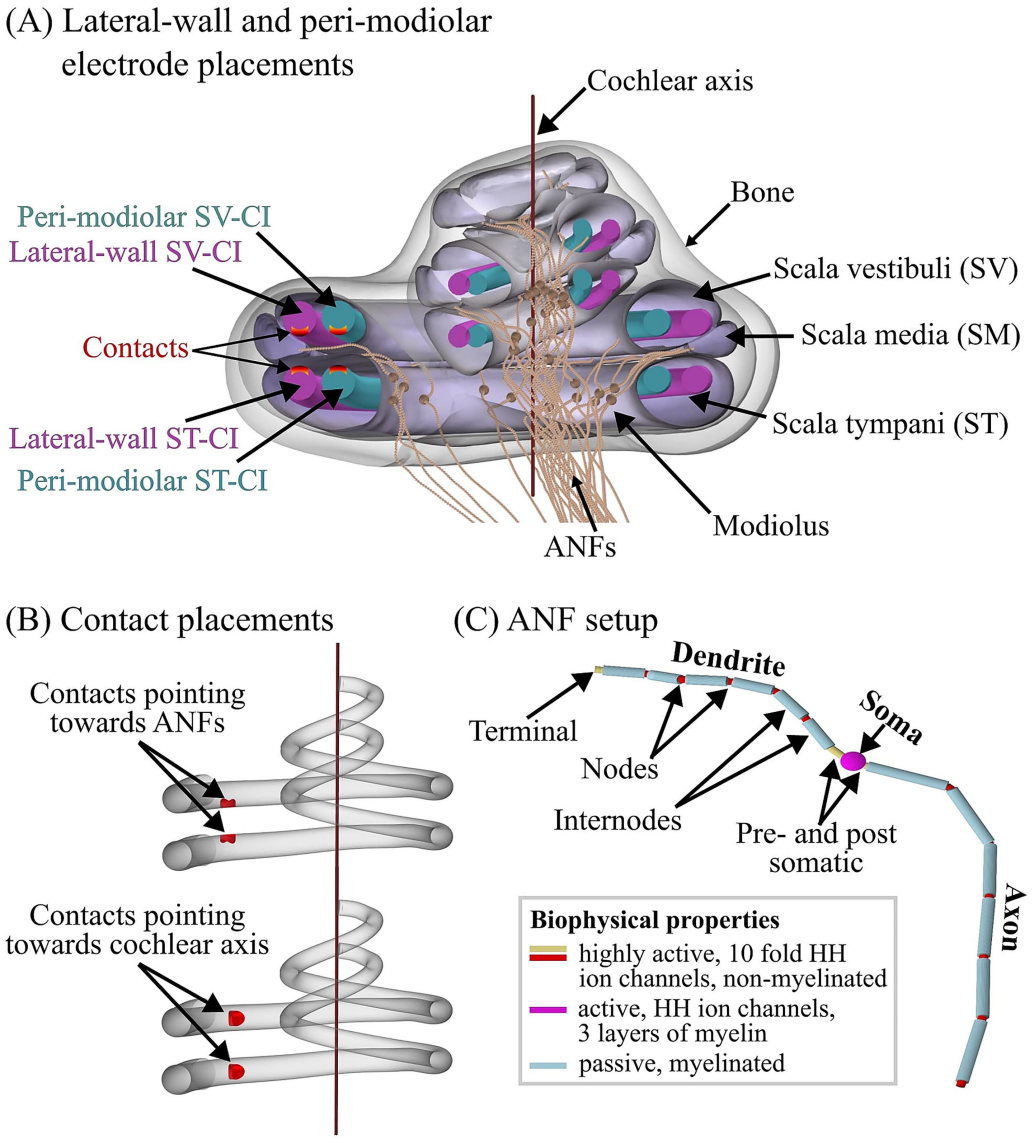
**(A)** Cross-section through the volume conductor model of the human cochlea showing five distinct electrical domains: scala tympani, scala vestibuli, scala media, bone, and the modiolus, which is transparent in this view. All four electrode arrays are shown for comparison. Soma positions are indicated as spheres on the brown ANFs. **(B)** Electrode contacts are oriented either toward the ANFs (top, default contacts) or toward the cochlear axis (bottom, alternative contacts). **(C)** Each ANF consists of myelinated segments (internodes) and segments with electrically active membranes marked in red, yellow, and purple. While the curvature of internodes is not depicted in the diagram, it was considered in the simulations. ANF: auditory nerve fiber; HH: Hodgkin-Huxley model.

A previously developed micro-CT segmentation of a human cochlea served as the foundation for generating the in-silico cochlear model (Potrusil et al., 2020). The final three-dimensional model comprises five distinct cochlear domains: the scala vestibuli (SV), scala tympani (ST), scala media, the modiolus (including the osseous spiral lamina), and the surrounding bone (Figure 1A). For subsequent finite element simulations, an additional spherical domain with a radius of 100 mm was added to enclose the entire cochlea. Further details regarding mesh generation and COMSOL geometry handling are provided in Fellner et al. (2024).

The in-silico model incorporates 25 anatomically accurate auditory nerve fiber (ANF) pathways, derived from a dataset of manually traced fibers (Potrusil et al., 2012; Fellner et al., 2024). Owing to the realistic nature of the dataset, the ANFs are not uniformly distributed along the cochlear spiral. Nevertheless, they provide anatomically precise trajectories (Figure 1A), spanning from the basal turn at *α* = 23° (ANF23) to the apical turn at α = 680° (ANF680), where α indicates the angle relative to the central cochlear axis (Figures 1A,B). Each ANF consists of a thinner peripheral process (diameter: 1.3 μm) and a thicker central process (diameter: 2.6 μm), which connect to the soma at their respective ends. The spherical soma (diameter: 20 μm; Potrusil et al., 2012) is connected to the processes by a 100 μm pre-somatic and a 5 μm post-somatic segment on the peripheral and central sides, respectively (Rattay et al., 2001b). Figure 1C provides a schematic representation of a single ANF with its various compartment types.

Due to the morphology of the human cochlea, the somas are distributed along Rosenthal’s canal, resulting in variable dendritic lengths. Specifically, the peripheral process lengths of the 25 ANFs range from 1,320 μm to 2,312 μm, with a median of 1,732 μm. Both the peripheral and central processes are composed of alternating sequences of myelinated internodes and electrically active nodes of Ranvier, each 2.5 μm in length. The standard internode length for the central process is set at 500 μm, which is twice the target length of the peripheral internode. The effective peripheral internode length for each ANF was determined based on the specific dendritic length and includes a 10 μm unmyelinated terminal region at the distal end, where a healthy ANF innervates an inner hair cell. As a result, the effective peripheral internode lengths of the 25 ANFs span from 233.6 μm to 274.5 μm, with a median of 253.5 μm, and the number of internodes varies from five (mostly in middle turn fibers) to nine (in two basal fibers).

To simulate different stages of neural degeneration, two conditions were modeled: severely degenerated ANFs, represented by a reduced dendritic diameter of 0.5 μm (Heshmat et al., 2020), and completely degenerated ANFs, which lack the entire peripheral process.

To complete the computational cochlear model in COMSOL, electrode arrays were incorporated as geometric entities. Each of the four arrays consists of a silicone carrier and electrical contacts (Figure 1A). The carrier was generated by extruding an array-specific centerline, with a diameter tapering from 0.6 mm at the basal start (*α* = 0°) to 0.3 mm at the apical end (α = 690°). The resulting length of the electrode arrays is approximately 25 mm. The contacts are modeled as cylinders positioned at the desired locations (specified by α and vertical or horizontal placement, Figure 1B).

For construction of the centerlines of each array (Figures 1A,B), both the ANF pathways and the geometries of the ST and SV were considered. The ANFs determined the slope of the arrays in the z-direction, while the canal geometries dictated the lateral positioning. An iterative algorithm employing cubic spline interpolation was used to ensure (a) z-aligned curves within the ST and SV that maintain an approximately constant minimum distance (255 μm) to all ANFs, and (b) lateral alignment of the lwST/lwSV and pmST/pmSV centerlines, with the narrower canal determining the position of both arrays to prevent intersection with any duct boundary.

### 2.2 Electrical parameters and model setup

The CI-induced extracellular potential (*Ve*) was evaluated using the Electric Current physics from the AC/DC module in COMSOL Multiphysics (Fellner et al., 2022). Default electric conductivities for various domains were selected based on previous studies (Rattay et al., 2001a; Potrusil et al., 2020), specifically: 1.43 S/m for the perilymph in ST and SV, 1.67 S/m in the endolymph-filled SM, 0.0334 S/m in the modiolus domain, 0.016 S/m for the compact bone and surrounding sphere, 1,000 S/m for the electrode contacts, and 0 S/m for the silicone carrier. Monopolar CI stimulation was simulated by setting a fixed current of 1 μA at an active electrode contact via the terminal boundary condition, while continuity was assumed across internal boundaries. The return electrode was implemented by assigning ground to the outer boundaries of the surrounding sphere (Fellner et al., 2024). Extracted V*_e_* values at the spatial coordinates of the 25 ANFs were used as inputs for subsequent analysis of excitation behavior in NEURON.

The electrical properties of the ANFs (Figure 1C) were modeled following the compartment model of a human type I spiral ganglion cell (Rattay et al., 2001b; Potrusil et al., 2020), with intracellular resistivity set to 0.1 kΩ·cm along the fiber. Membrane capacitance and conductance of the internodes were considered inversely proportional to the number of membrane sheets (1 μF/cm^2^ and 0.1 mS/cm^2^ per sheet), with the dendrite modeled with 40 sheets and the axon with 80 (Arnesen et al., 1978; Arbuthnott et al., 1980; Spoendlin and Schrott, 1989; Rattay et al., 2002). Ion channel kinetics followed a temperature-adjusted Hodgkin Huxley model aligning with reported action potential (AP) duration in the human cochlea (Rattay et al., 2013). For the soma of human ANFs, three surrounding membrane layers were included (Schnabl et al., 2012). In other electrically active compartments—such as the nodes of Ranvier, pre- and post-somatic regions, and the peripheral terminal—a tenfold increase in Hodgkin Huxley membrane conductance was applied to reflect experimentally observed high ion channel density (Rattay et al., 2002; Hossain et al., 2005).

The investigation examined excitation profiles resulting from monophasic stimulation, specifically analyzing 50 μs long monophasic pulses for cathodic stimulation (results sections) and comparing them to anodic stimulation results (discussion section 4.3), unless otherwise noted. Additional information regarding the NEURON implementation and threshold search algorithms is available in Fellner et al. (2024).

## 3 Results

### 3.1 Cathodic threshold stimulation of lateral-wall and peri-modiolar electrode arrays in ST and SV

**Figure 2:**
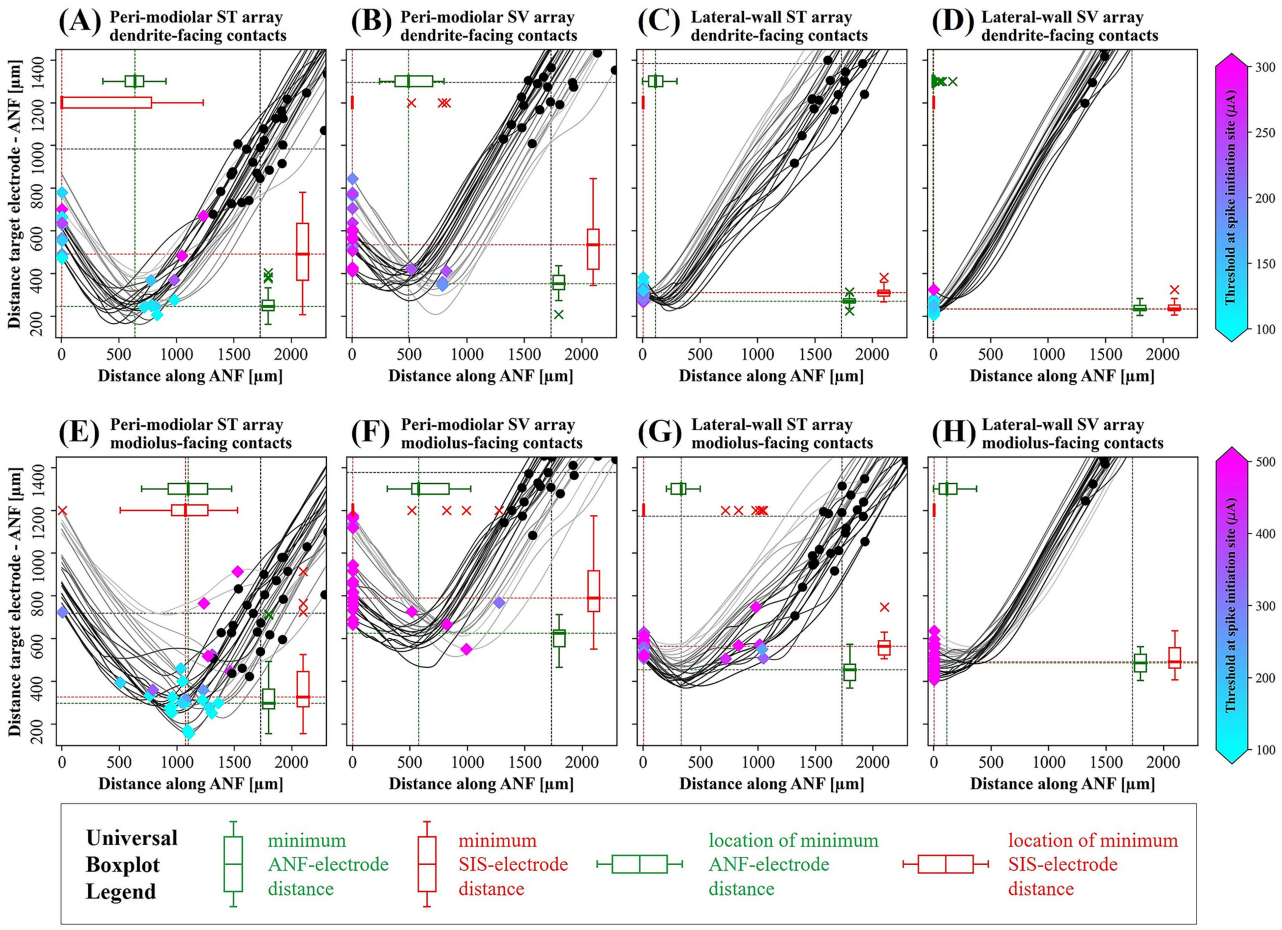
Electrode contact–fiber distance relationship and cathodic threshold stimulation site. Upper panels show results for default contacts facing upward in the ST and downward in the SV: **(A)** pmST, **(B)** pmSV, **(C)** lwST, **(D)** lwSV. Lower panels show results for alternative contacts facing the cochlear axis: **(E)** pmST, **(F)** pmSV, **(G)** lwST, **(H)** lwSV. In each panel, lines represent the distance to the target contact for the first 2,400 μm of each ANF, with light to dark gray colors indicating basal to apical ANFs. Somas within 1,450 μm of the target contact are marked by black dots, and the corresponding median soma–contact distance is plotted as a black dotted horizontal line. The black dotted vertical line indicates the median soma position (peripheral process length), which is consistent across panels. The green vertical boxplot at the lower right shows statistics for the minimum contact–ANF distance among all fibers, with its median highlighted as a green dotted horizontal line. Statistics for the site of minimal distance are represented by horizontal green boxplots at the upper left, with the green dotted vertical line indicating the median. The SIS is marked by diamonds, with color indicating the absolute cathodic threshold according to the color bars for upper and lower panels. SIS and its distance to the target contact are represented by red boxplots and dotted lines. ANF: auditory nerve fiber; SIS: spike initiation site.

The placement of the four arrays results in different electrode-to-ANF distance profiles. In Figure 2, gray lines show these distances along the first 2,400 μm of each ANF, with shading from basal (*α* = 23°, ANF23) to apical (ANF680) fibers. Dendritic lengths vary, so each soma is at a different distance from the electrode. The median dendritic length is marked with a vertical black dotted line. Somas less than 1,450 μm from the electrode are highlighted with black dots. For the pmST array (Figure 2A), all somas are visible with a median soma-electrode distance of 983 μm. The pmSV (Figure 2B) and lwST (Figure 2C) arrays show median distances of 1,294 μm and 1,384 μm, respectively. Only a few somas are visible for lwSV (Figure 2D), with its median distance exceeding the scale.

For pm arrays the minimum electrode-to-ANF distance typically occurs at a mid-dendritic region, while lw electrodes are positioned closest to the dendritic terminal (green boxplots). The median minimum distances are 246 μm (pmST), 351 μm (pmSV), 269 μm (lwST), and 233 μm (lwSV).

During monophasic cathodic stimulation, the region of the ANF nearest the electrode is depolarized, while adjacent compartments become hyperpolarized. This creates an oscillating transmembrane potential profile along the ANF, identifying possible spike initiation sites (SIS’s), depicted as diamonds in Figure 2. Blue diamonds indicate thresholds around 100 μA, while magenta diamonds indicate thresholds of 300 μA or higher. The red horizontal boxplots in each panel summarize SIS location statistics, with the median marked by a red vertical dotted line, and the vertical red boxplot at the lower right showing the distribution of electrode-to-SIS distances.

For lwSV contacts, the AP is always initiated at the peripheral terminal, with a median SIS-electrode distance of 233 μm, as the electrode is nearly always closest to the terminal, resulting in direct depolarization and minimal latency. In lwST, the minimum electrode-ANF distance shifts slightly away from the terminal, causing depolarization at the first peripheral node. However, the SIS remains the terminal, as intra-cellular current from the depolarized neighbor and higher ion channel density favor this site, resulting in a slight delay in AP onset compared to lwSV.

For the pmSV array, the minimum electrode-ANF distance is further along the dendrite. Although maximum depolarization is observed at the third peripheral node during pulse application, the AP typically starts at the sodium channel band in the terminal due to intracellular current flow. This retrograde propagation of intracellular current results in a modest delay in the initiation of the AP. For pmST, ten ANFs have SIS between the third and fifth peripheral node, while the remainder initiate spikes at the more distant terminal, also with higher latency.

When electrode contacts are repositioned around the array carrier to face the modiolus (Figures 1B, 2E–H), the minimum electrode-ANF distance increases for all arrays: the median minimum distances are 297 μm (pmST), 625 μm (pmSV), 453 μm (lwST), and 487 μm (lwSV). Notably, for the pmST array, three fibers exhibit a reduced minimum electrode-ANF distance compared to dendrite-facing contacts. The site of minimum electrode-ANF distance is also further from the terminal for all arrays in this configuration.

As a result, absolute thresholds (color-coded diamonds in bottom panels of Figure 2, scale 100–500 μA) and SIS locations shift in some cases. For pmST, SIS typically moves from the terminal to the second node through the pre-somatic compartment, with only ANF195 retaining terminal initiation. The median SIS location for pmST is now 1,075 μm from the terminal. For the other arrays, most fibers still initiate at the terminal, though a few pmSV and lwST fibers show shifted SIS. For the lwSV array with modiolus-facing contacts, the SIS remains the terminal for all fibers. Only in pmST the median SIS-electrode distance decreases compared to the dendrite-facing contact.

**Figure 3:**
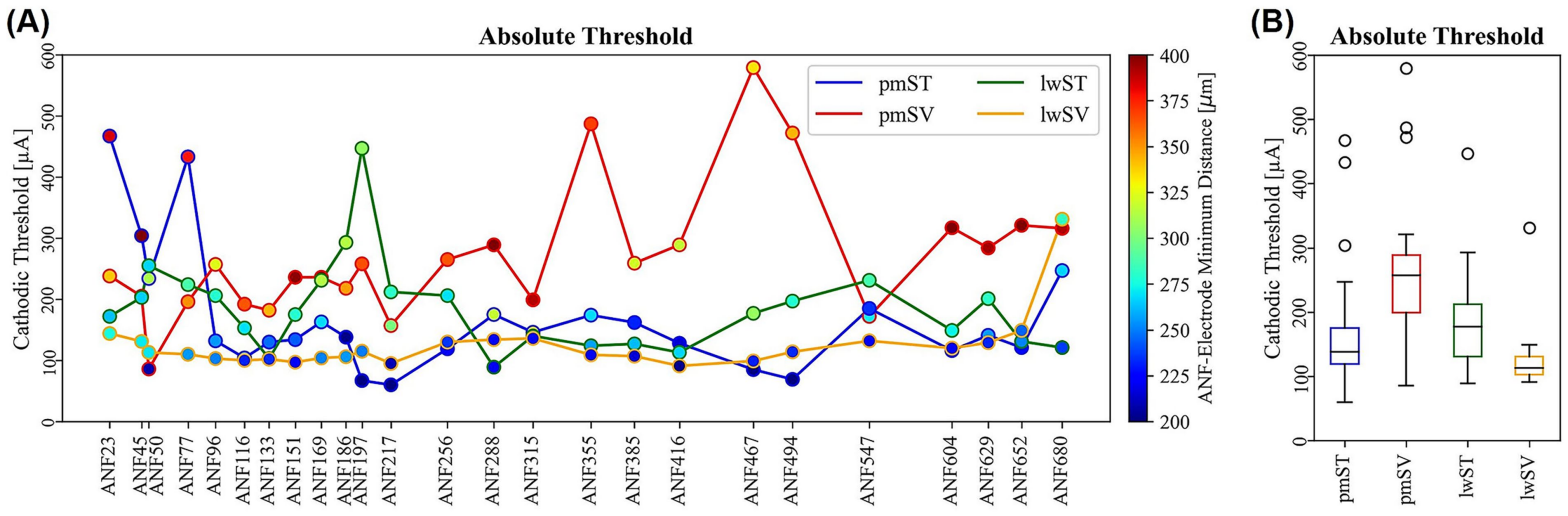
Absolute monophasic cathodic thresholds for four electrode arrays and 25 target ANFs. **(A)** Line plot showing the threshold in μA (y-axis) for each ANF (x-axis). An additional scatter plot presents the respective minimum distance in μm between electrode contact and target ANF, as indicated by the color bar on the right. **(B)** Boxplots of cathodic thresholds for different electrode arrays. ANF: auditory nerve fiber.

Thresholds vary among ANFs and electrode arrays. Figure 3A presents a comparison of absolute thresholds across all ANFs, with distinct line colors representing each of the four electrode arrays (refer to the legend in Figure 3A). The color of the filled circles denotes the minimum electrode-ANF distance in μm for each fiber and its corresponding target electrode contact (see the color bar on the right in Figure 3A). Notably, lower minimum distances are observed for pmST and lwSV (blue filled circles), whereas lwST and pmSV demonstrate higher values (green and orange/red filled circles). There is a strong positive correlation between threshold and minimum electrode-ANF distance for pmST (Pearson r = 0.84). The correlation is weaker for lwST (0.72), lwSV (0.51), and pmSV (0.33), but remains positive.

As shown in Figure 3B, the lowest median cathodic threshold is for lwSV (113 μA), which also has the least variation. pmST has the second lowest median (138 μA), affected by three high basal outliers (Figure 3A). The medians for lwST and pmSV are 177 μA and 257 μA, respectively (Figure 3B), with only the pmSV contact at 50° showing increased sensitivity (Figure 3A).

These results indicate that, in general, ST thresholds are higher than SV thresholds for lw arrays, while the opposite is true for pm arrays. Comparing pm and lw positions, pm arrays have lower thresholds for ST, while lw arrays have lower thresholds for SV.

With alternative contact positions, the median cathodic threshold for lwSV rises to 495 μA, a 340% increase over the default configuration. This is due to the greater distance from the alternative contacts, while the SIS remains at the terminal. Thresholds also increase for pmSV and lwST with alternative contacts, with new median values of 451 μA (83% increase) and 315 μA (70% increase), respectively. In contrast, for the pmST array the SIS shifts closer to the site of minimum electrode-ANF distance, which results in a lower median threshold of 116 μA (18% decrease compared to the default).

Thus, for electrode contacts oriented toward the cochlear axis, pmST thresholds are typically the lowest, followed by lwST, with pmSV or lwSV showing the highest values. In this case, ST arrays generally have lower thresholds than SV arrays for both lw and pm placements. As with the default setup, lw thresholds are usually higher than pm thresholds for ST, while SV thresholds are similar for pm and lw arrays with alternative contacts.

### 3.2 Comparing threshold excitation for healthy and degenerated ANFs

**Figure 4:**
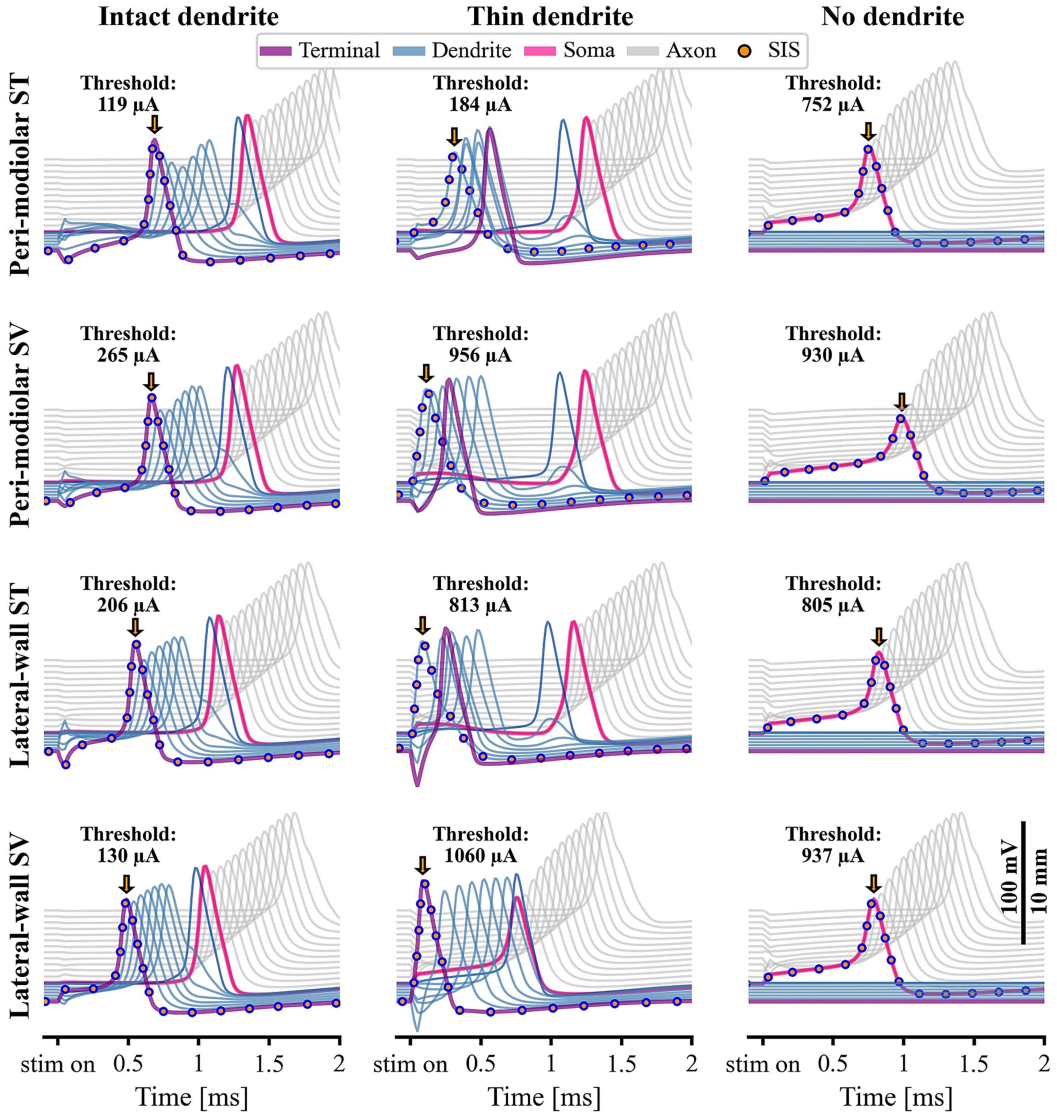
Propagating action potentials (APs) at cathodic threshold stimulation for ANF256 modeled as intact neuron (left column), severely degenerated (thin dendrite, middle), and completely degenerated (no dendrite, right). Results for the target electrode contact at different array locations are shown in rows. Each line plots the transient transmembrane voltage within one active section, with different colors corresponding to compartment types (see legend at the top). For clarity, the lowest magenta line indicates the terminal, and results are restricted to 12 axonal nodes. The section where the AP is elicited, the SIS, is highlighted by a dotted line, and peak amplitudes are marked with orange arrows. The scale bar at the bottom right shows transmembrane voltage and compartment distance shift. ANF: auditory nerve fiber; AP: action potential; SIS: spike initiation site.

Figure 4 summarizes the propagation of APs in ANF256 following cathodic threshold stimulation by the four electrode arrays (rows), comparing three fiber conditions: a healthy ANF with an intact dendrite (1.3 μm diameter; left column), a severely degenerated ANF with a thin dendrite (0.5 μm; middle), and a fully degenerated ANF without a dendrite (right). In each panel, lines trace the transient transmembrane voltage across active compartments, from the terminal (purple, bottom), through seven peripheral nodes (blue), the soma (pink), to the central nodes (grey, top). For clarity, pre- and post-somatic compartments are omitted and only 12 axonal nodes are depicted. The vertical displacement reflects the physical separation between compartments, as shown in the scale bar in Figure 4. The SIS is identified by the earliest AP peak (orange arrows) and highlighted with dots.

In healthy ANF256, the AP consistently initiates at the terminal, even when maximal depolarization occurs nearer to the peripheral nodes (except for lwSV, where the terminal is directly depolarized at the site of minimal electrode-fiber distance). For other arrays, the minimum electrode-ANF256 distance, and thus the region of strongest depolarization, is further from the terminal. Nevertheless, the terminal is triggered first via intercellular current flow, resulting in minor delays in AP onset (orange arrows). The absolute cathodic current required for AP initiation varies slightly among the arrays, with shorter electrode-fiber distances corresponding to lower thresholds. In all four arrays, the AP propagates in the typical pattern: from the terminal through peripheral nodes with comparable peak amplitudes, followed by a somatic delay due to the soma’s high capacitive load.

When the dendritic diameter is reduced (middle column of Figure 4), intra-cellular current flow decreases, challenging transmission over the unchanged soma. Despite this, electrode-ANF distance relationships persist, and active contacts remain closer to the dendrite rather than the axon. Consequently, the SIS shifts to the site of initial depolarization, coinciding with the minimal electrode-ANF distance: at the terminal for lwSV, the first peripheral node for lwST, the second for pmSV, and the third for pmST. Only for pmST does the threshold (184 μA) remain comparable to the healthy fiber; thresholds increase markedly for the other arrays.

For pmST, pmSV, and lwST, the SIS shift to regions of direct depolarization results in earlier AP onset. The AP propagates toward the central end with a somatic delay and also back-propagates to the terminal. For lwSV stimulation of a thin dendrite, the higher current required to trigger the AP in the closely positioned terminal causes the spike to occur nearly immediately after the pulse, with no somatic delay. The AP proceeds along the dendrite, and a spike is simultaneously triggered in the soma, albeit with a lower peak amplitude, still sufficient for successful propagation. The fastest conduction is observed for lwSV threshold stimulation of the thin dendrite.

In completely degenerated ANF256 (right column of Figure 4), AP initiation occurs via direct depolarization in the soma for lw arrays, or the short post-somatic compartment for pm arrays. Note that the distance between the centers of soma and post-somatic compartment is only 12.5 μm and the corresponding voltage-profiles are not discernable in Figure 4. Thresholds correlate with electrode-soma distance: pmST, being closest, requires the lowest threshold, and lwSV, being most distant, requires the highest. Notably, fully degenerated fibers can have a lower cathodic threshold than severely degenerated fibers, and central spike arrival occurs earlier. Sites of significant depolarization also appear in axonal nodes, especially with SV arrays, and in several of the investigated cases (not shown), especially for supra-threshold stimulation, the SIS shifts to axonal nodes.

**Figure 5:**
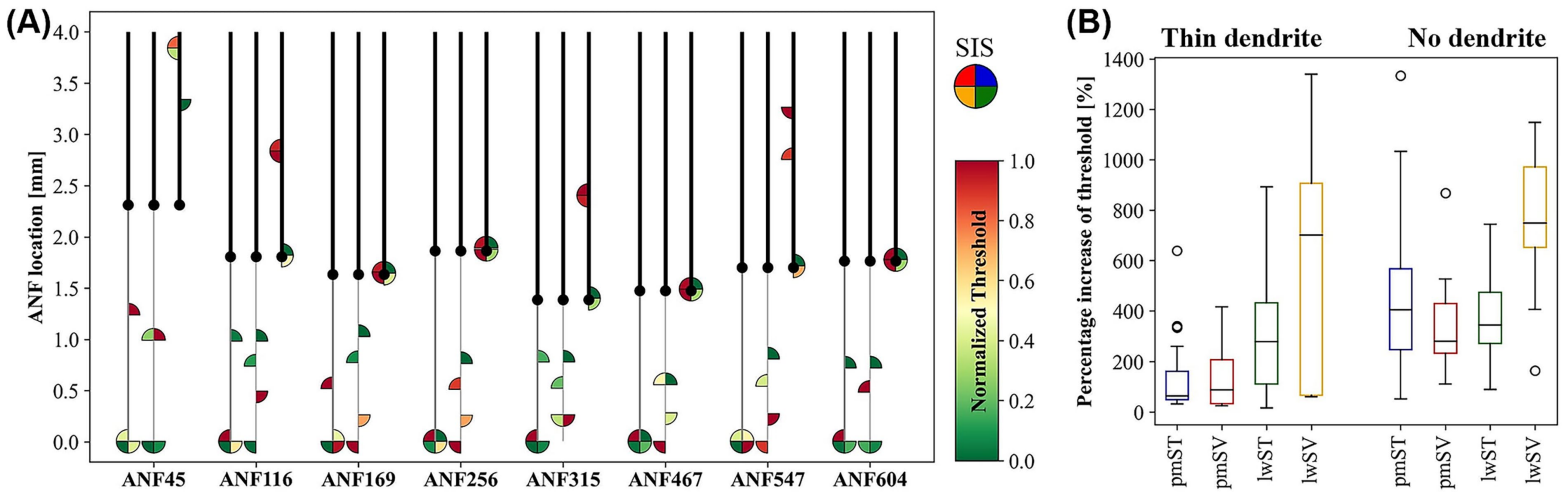
**(A)** Schematic of selected ANFs depicted as straight fibers. For each fiber, the healthy (left), severely degenerated (thin dendrite, middle), and completely degenerated (no dendrite, right) cases are shown side by side. The SIS for each of the four electrode arrays is marked as a quarter-filled circle at the corresponding position along the ANF: pmST (upper right), pmSV (upper left), lwST (lower right), lwSV (lower left). The color of the SIS circle represents the normalized threshold (color bar at right), with normalization performed for each ANF and neural status. **(B)** Percentage increase of cathodic thresholds in all ANFs with increasing degree of degeneration. ANF: auditory nerve fiber; SIS: spike initiation site.

Figure 5A compares healthy and degenerated fibers for eight selected ANFs, including ANF256, depicted as straightened fibers. For each ANF, the three conditions are shown from left to right: healthy, thin dendrite, and no dendrite. The SIS is marked as a filled quarter circle, with each quadrant corresponding to a specific electrode array. The color indicates the normalized threshold: dark red for maximum and dark green for minimum within the fiber.

Consistent with Section 3.1, in healthy ANFs (left), the SIS is always at the terminal for lw arrays and often for pm arrays. Dendritic SIS shifts are observed for some healthy ANFs with pmST stimulation, and color ranking indicates that lwSV positions have the lowest thresholds, while pmSV typically exhibits the highest.

For thin dendrites (middle), the SIS frequently shifts to the peripheral site closest to the electrode; only the lwSV array consistently retains the terminal as SIS due to proximity to contacts. Thresholds increase in all arrays compared to healthy ANFs, with the smallest average increase (63%) in pmST, and the largest (701%) in lwSV. Increases for lwST and pmSV are 278 and 87%, respectively. Thus, pm arrays are less affected by early degeneration, with lowest thresholds for pmST, followed by pmSV, then lwST, and highest for lwSV. ST thresholds exceed those of SV only in the most basal ANFs; otherwise, ST thresholds remain lower for both pm and lw arrays.

In fibers lacking dendrites (right), APs are typically initiated in the soma or post-somatic region, with occasional initiation in central nodes. Thresholds rise further, with the smallest average increase (280%) in pmSV, followed by pmST (406%) and lwST (345%). For lwSV, the average increase (748%) is similar to that for thin dendrites. The lowest median threshold is observed for pmST, with lwST about 12% higher. The highest thresholds are nearly equal for pmSV and lwSV, on average 39 and 24% higher than their ST counterparts.

### 3.3 Co-stimulation, selectivity, and specificity of different electrode arrays

Stimulation of non-target fibers, referred to as co-stimulation, has been observed in both clinical and computational studies, while cross-turn stimulation describes activation of fibers from different cochlear turns (see, e.g., Croner et al., 2022). To evaluate the selectivity and specificity of the four electrode arrays, we determined cathodic thresholds for all 25 ANFs across each of the 25 electrode contacts. Regardless of electrode array position, the highest absolute cathodic thresholds—up to 2 mA—were recorded when stimulating with the most basal electrode (EL23) and fibers located roughly 180° opposite in the cochlea. Similarly, high thresholds were needed for electrode EL197 to excite ANF23. Elevated thresholds were also common for neighboring fibers just preceding the most apical fibers when stimulated by corresponding apical electrodes.

For cathodic threshold stimulation, the SIS is consistently at the terminal for lateral wall (lw) arrays and typically for the pmSV array. For the remaining pmSV cases and about 60% of target fibers stimulated by the pmST array, the SIS shifts to peripheral nodes. Neighboring fibers often initiate APs at their terminal or a peripheral node, especially with ST arrays. For fibers beyond the target and its immediate neighbors, the SIS generally shifts to the pre-somatic compartment. Axonal initiation sites are rare, observed only in neighboring fibers of basal ANFs and in a few cases among lower neighbors of the most apical ANFs.

**Figure 6:**
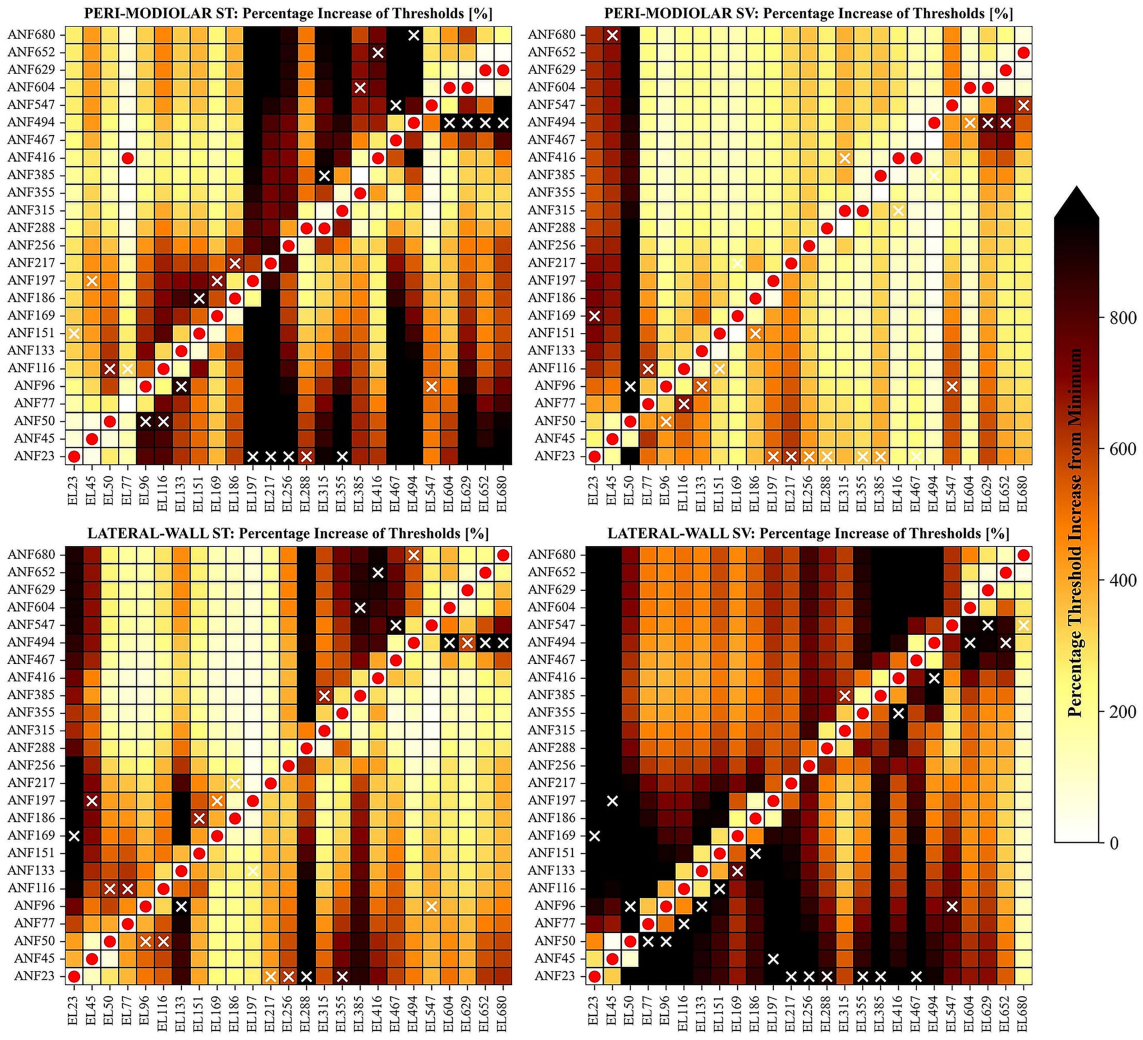
Heatmaps of percentage increase in minimum thresholds for all ANFs (rows) and all electrode contact positions (columns). Results are shown in separate panels for pmSV (top left), pmSV (top right), lwST (bottom left), and lwSV (bottom right) arrays. For each electrode position, the fiber with the minimum cathodic threshold is marked by a red dot; the fiber with the maximum threshold is marked by a white cross. Pixel color indicates the percentage increase in cathodic threshold compared to the minimum threshold for each electrode contact position, as shown in the color bar at right. ANF: auditory nerve fiber.

To quantify array specificity, we evaluated normalized thresholds for all fibers with each electrode contact, identifying how many additional fibers are activated by a pulse near the target threshold. Figure 6 shows the percentage increase from the minimum threshold for each electrode position, highlighting the range between minimum and maximum thresholds. Yellow pixels indicate a 200% increase, while black denotes thresholds about ten times higher than the minimum. The minimum (red dots) and maximum (white crosses) thresholds for each contact are also marked. Notably, both pm arrays display some irregularities in minimum thresholds, especially above 315°, where the fiber before the target sometimes exhibits a lower threshold. In the most apical fibers, thresholds can be 2–3 times higher than the lowest threshold recorded in their neighbors. For instance, electrode contact at 77° in pmST yields a slightly lower threshold for ANF416 than for its target ANF77, located nearly one turn (360°) below. Generally, maximum thresholds for a given contact are found among immediate neighboring ANFs, but ANF23 and ANF494 frequently show the highest thresholds, making them the hardest to excite regardless of array location.

Median percentage increases from minimum threshold for each electrode array are: 473% for pmST, 277% for pmSV, 370% for lwST, and 635% for lwSV. Thus, under cathodic stimulation, lwSV demonstrates the highest specificity, as reflected by its heatmap showing significantly elevated thresholds for non-target fibers. The pmST array exhibits higher specificity in middle and apical turns, with similar patterns seen for pmSV and lwST.

To assess selectivity, thresholds were normalized for each ANF across all electrode contacts. Similar irregularities were observed, with both pm arrays sometimes showing that the electrode after the target contact induces a lower threshold for fibers above 288°. For lwST stimulation of ANF197, the threshold at the target contact (EL197) was slightly higher than at EL315. Across all arrays, maximum thresholds were almost always recorded at EL23. Regions of lowest selectivity varied by array: in pmST, the most basal fibers; in pmSV, fibers around *α* = 450–500°; in lwST, around 200°; in lwSV, around 300° and the most apical fiber. Nevertheless, the overall trend persists, with lwSV exhibiting the highest median threshold increase (635%), followed by pmST (467%), lwST (339%), and pmSV showing the lowest selectivity (281%).

### 3.4 Variation of electrical conductivities and ST ossification

**Figure 7:**
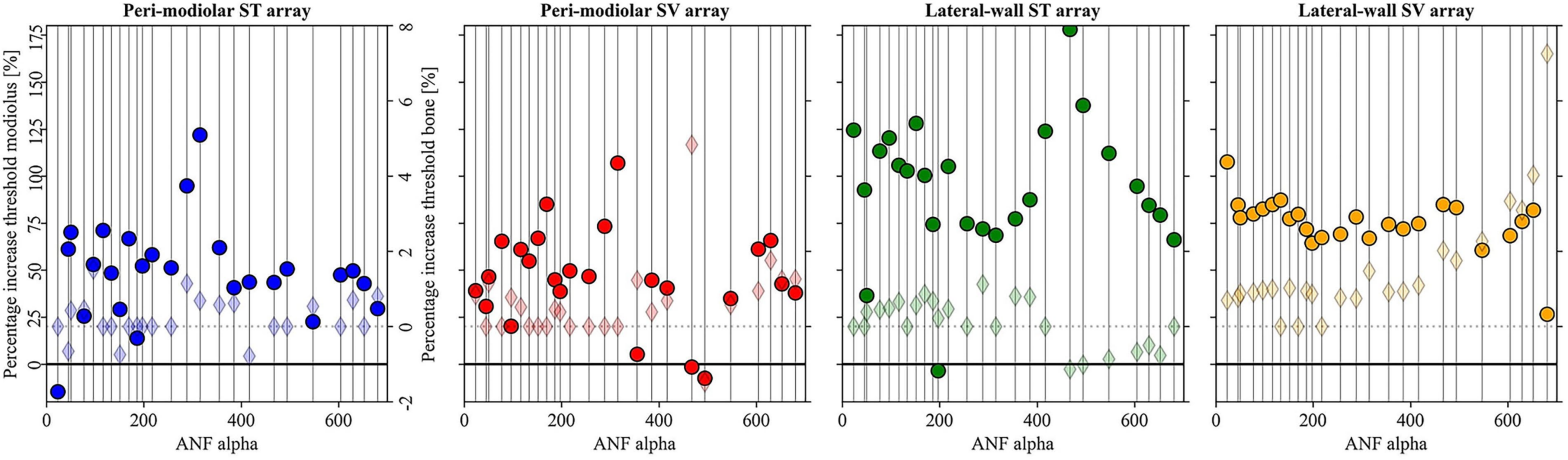
Percentage increase of cathodic threshold in all fibers (x-axis) for pmST (first panel), pmSV (second panel), lwST (third panel), and lwSV (fourth panel) arrays under two specific conductivity variations. Results for a 10-fold increase in modiolus conductivity are shown with filled circles and correspond to the left y-axis in each panel. The lower percentage threshold increase for a twofold increase in bone conductivity is indicated by filled diamonds, corresponding to the right y-axis. Y-axis scales are consistent across panels for direct comparison.

Figure 7 illustrates the percentage increase in cathodic thresholds across all 25 ANFs (x-axis) with elevated conductivities in the modiolus (filled circles, primary y-axis) and bone (filled diamonds, secondary y-axis). When modiolus conductivity is increased tenfold, less current accumulates within the ANF domain, reducing induced extracellular potentials along the fibers and generally increasing cathodic thresholds. Apical ANFs appear less affected, and median threshold increases are less pronounced for pm arrays (averaging 50% for pmST and 45% for pmSV) than for lw arrays (95% for lwST and 77% for lwSV). Although the relative ranking of array thresholds remains unchanged, median values for pmST (202 μA) and lwSV (198 μA) are now similar and about half the median values observed for pmSV (389 μA) and lwST (371 μA).

In certain cases, thresholds decrease. For example, when ANF23 is stimulated by its target pmST contact (blue filled circle), the SIS shifts from the terminal to the third central node. Slightly lower thresholds are also observed for ANF467 and ANF494 (pmSV, red filled circles) and ANF197 (lwST, green filled circle) under increased modiolus conductivity, where the SIS remains at the terminal. The pmST array exhibits the most SIS changes and is the only array to retain some peripheral AP initiation sites at higher modiolus conductivity. For pmSV and lwST, APs are predominantly initiated at the terminal, with only rare central node initiation at high thresholds. The lwSV array demonstrates the most uniform threshold increase, with the SIS consistently remaining at the terminal.

When bone conductivity is increased, the cochlea’s outermost layer becomes less resistive, allowing more current to flow into the surrounding bone. However, the effect on the cochlear interior is minimal, leaving induced extracellular potentials along the ANFs largely unchanged. With doubled bone conductivity thresholds remain nearly unchanged, though apical fibers show a slight, nearly exponential increase, particularly in the lwSV array (light orange filled diamonds). Even in lwSV, the highest increase (in ANF680) is less than 8%, and the median percentage increase is negligible for all arrays. SIS changes with altered bone conductivity are rare: none for lw arrays and only minor terminal-to-peripheral node shifts for pm arrays.

Simultaneously increasing modiolus and bone conductivities to 0.334 and 0.032 S/m, respectively, results in an approximate accumulation of the individual effects (data not shown).

**Figure 8:**
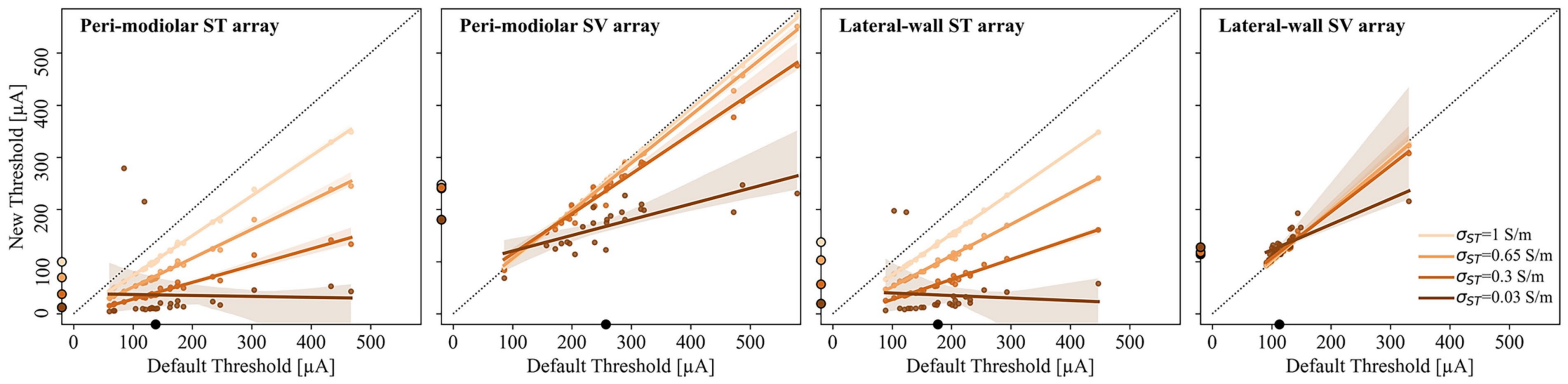
Scatter plots of default (x-axis) versus new threshold (y-axis) for simulations with varying degrees of ST ossification. Results for pmST, pmSV, lwST, and lwSV arrays are arranged from left to right. Different colors indicate specific ST conductivity values, ranging from 1 (developing ossification) to 0.03 S/m (complete ossification), according to the legend in the right panel. For each point cloud, the linear regression line and corresponding confidence interval are shown in matching colors. The diagonal dotted black line indicates the boundary between new lower thresholds (most common, below the line) and new higher thresholds (above the line). Median threshold values are indicated by dots on the x-axis for the unobstructed ST and on the y-axis for varying degrees of ossification. X- and y-axis scales are fixed across all panels for comparison.

Ossification was evaluated by decreasing ST conductivity. Figure 8 presents scatter plots comparing default thresholds (x-axis) to those for altered ST conductivities: 1 (developing ossification), 0.65 (moderate), 0.3 (severe), and 0.03 S/m (complete ossification). Median thresholds are shown as filled circles, with linear regression lines and confidence intervals (legend in right panel). It should be noted that severe to complete ossification precludes ST insertion; thus, these results are hypothetical.

For pmST stimulation, median thresholds decrease from 100 to 13 μA as ossification progresses, with an average reduction of 26% (developing), 48% (moderate), 71% (severe), and 91% (complete ossification). Minor SIS shifts to peripheral initiation sites occur in about one-third of ANFs at early ossification, affecting half of ANFs at complete ossification. Similar trends are observed for lwST (median thresholds from 138 to 20 μA), with an average decrease only 2% less than pmST. The linear relationship between patent and ossified thresholds is maintained, with greater decreases at higher thresholds. Outliers for increased thresholds differ between arrays. No SIS shifts are seen for lwST, where APs are almost always terminal-initiated even in ossified ST.

For the pmSV array, median thresholds remain around 243 μA from developing to severe ossification which is slightly lower than the default threshold of 257 μA. For complete ossification the median threshold decreases to 181 μA (31% reduction). The linear relationship persists, though less pronounced, with broader confidence intervals. Up to a quarter of ANFs (*α* < 170°) show peripheral SIS shifts at all ossification stages.

For lwSV, median thresholds remain stable (113 μA default, 114 μA to 118 μA for developing to severe ossification), with only a 4% increase. At complete ossification, the median is 128 μA (10% increase). A single outlier (ANF680) causes a wider confidence interval, but otherwise, thresholds correspond closely for all ossification stages. In the case of lwSV, threshold increases exhibit a linear decline from basal to apical ANFs, ultimately resulting in threshold decreases for the most apical fibers. Across all ST conductivity assumptions, the SIS remains at the terminal due to its proximity to the stimulating electrode and high ion channel conductance.

In summary, increased modiolus conductivity raises thresholds by reducing extracellular potentials, while bone conductivity changes have minimal impact. ST ossification lowers thresholds for ST arrays (pmST, lwST) but not for SV arrays (pmSV, lwSV), where SIS and thresholds remain stable.

## 4 Discussion

### 4.1 Cathodic threshold excitation behavior in different electrode array locations

In this study, we investigated monopolar CI stimulation using short monophasic cathodic pulses, following the approach of Miller et al. (1999). This methodology avoids biphasic pulses and bipolar electrodes, which can produce complex, time- and space-varying excitation patterns that complicate data interpretation. To assess key differences in excitation behavior, we constructed electrode arrays positioned equivalently along scala tympani and scala vestibuli where we maintain consistent distances between active contacts and target auditory nerve fibers (ANFs). While this design does not replicate the electrode configuration of clinically utilized CIs—which typically have fewer contacts at fixed intervals (Dhanasingh and Jolly, 2017; Zeng et al., 2008)—it enables comparison of outcomes across different, realistic ANF pathways.

Cathodic thresholds for AP initiation varied across ANFs and electrode arrays (Figures 2, 3). Among healthy fibers, median thresholds ranked lowest to highest for lwSV, pmST, lwST, and pmSV arrays. Specificity demonstrated a similar ranking, with the lwSV array producing the largest threshold increases in non-target fibers (Figure 6). These findings align with Mistrík et al. (2017), who suggested that cochlear anatomy may cause significant frequency mismatches for apical electrodes in pm arrays, potentially impairing speech perception. Additional evaluations with a homogenous point source model revealed that geometric relationships between ANFs and electrode contacts strongly influence the observed behavior. For example, the observation that ANF23 frequently requires the highest current to be activated by certain electrode contacts can be attributed to its spatial positioning.

For cathodic threshold stimulation of healthy target ANFs and their immediate neighbors, the SIS was typically the dendritic terminal or a peripheral node. In other co-stimulated fibers, the SIS shifted to the pre-somatic compartment, with axonal SISs occurring only rarely. Similar patterns have been reported in studies using either the same (Fellner et al., 2024; Heshmat et al., 2020; Potrusil et al., 2020) or different neuron models (Kalkman et al., 2022). Detailed comparisons between neuronal excitation models are available in Bachmaier et al. (2019) and Bai et al. (2020).

To evaluate the impact of geometric neuron parameters on threshold excitation, we tested a halved peripheral internode length (125 μm) and a fixed dendritic length at the median value (1.7 mm). In both scenarios, average threshold changes were negligible (<5%), though some ANFs, especially in lwST and pmSV arrays, showed more substantial increases or decreases. These findings indicate that such model assumptions do not significantly affect threshold variation among ANFs.

Although our threshold currents and their variations are comparable to those observed in intracochlear single fiber stimulation experiments (Miller et al., 1999), the variations along the insertion angle *α* (Figure 3) are larger than those found in computational studies with linearly aligned neurons (e.g., Kalkman et al., 2014). This is attributed to realistic, irregular ANF pathways from μCT data (Potrusil et al., 2020) and fan-shaped distal endings causing variable electrode-terminal distances, especially for pm arrays (Figure 2). Similar spatial irregularities and their effects on excitation were modeled stochastically by Bai et al. (2019).

It is important to emphasize that the specific relationship between electrode and target ANF distance critically determines AP initiation sites and absolute threshold values. Prior work on lw arrays placed in ST and SV (Fellner et al., 2024) showed that even small shifts in stimulating electrode position can substantially affect thresholds in target ANFs. In this study, we further analyzed the effect of changing the electrode angle around the CI carrier (Figures 2D–G), repositioning contacts from upward/downward orientation to facing the cochlear axis (Figure 1B). Increased electrode-fiber distances in excitation-sensitive regions led to higher cathodic thresholds for all arrays except pmST. This increase can be significant, shifting lwSV from the most to least favorable array in terms of cathodic threshold amplitude. Notably, a common clinical electrode setup corresponds to pmST with contacts facing the cochlear axis, which is a reliable though not always optimal choice according to our results. Additionally, only MedEL CIs feature pairs of opposite-side electrode contacts at certain locations, making them the sole clinically used implants capable of inducing stimulation in the directions modeled here. This versatile technology appears especially beneficial for lw electrode arrays and SV insertion.

### 4.2 ANF degeneration

The dendrites of auditory nerve fibers (ANFs) degenerate earlier than their soma–axon connections, both in normal human ears and in cases of hearing loss that exceed age-related norms. In a study of 62 normal-aging human ears, Wu et al. (2023) found that ANF degeneration progresses significantly faster in the basal than in the apical half of the cochlea. Reductions in dendrite diameter and myelin thickness correlate with the degree of hearing impairment (Heshmat et al., 2020). The distribution of dendrite diameters shifts from a Gaussian-like profile in normal hearing or mild loss to a bimodal pattern in more severe deficits, with a subgroup of thin dendrites (0.3–1 μm, peaking at 0.5 μm). Consequently, many CI users, especially those with lw arrays, can be expected to have fewer focally activated ANFs in high-frequency regions. Some users therefore receive the same stimulus on adjacent contacts to activate a larger ANF population at sites of poor auditory perception (Hochmair et al., 2015).

Computational modeling studies have shown that reductions in dendritic diameter have a greater impact on threshold increases than decreases in myelin thickness (Wenger et al., 2023; Heshmat et al., 2020). Therefore, this study held myelination constant and simulated two scenarios: one with thin dendrites (d = 0.5 μm) representing severe degeneration, and one without dendrites to represent complete degeneration.

Consistent with prior findings, our results predict increased cathodic thresholds for degenerated ANFs. For thin dendrites, the smallest and largest threshold increases were observed for pmST and lwSV arrays, respectively (Figure 5B). As a result, pm thresholds are generally lower than lw thresholds in both cochlear canals, and SV thresholds are only lower than ST thresholds for the most basal ANFs.

With thin dendrites, the SIS often shifts away from the terminal, except for lwSV (Figures 4, 5A). This explains the substantial increase in threshold: thinner dendritic terminals require higher currents for successful AP propagation over the soma. Additional simulations indicated that a dendritic diameter of about 0.65 μm eliminates drastic threshold increases across all fibers and array locations. For example, in the case of ANF256, increasing the dendrite diameter from 0.5 μm to 0.6 μm substantially lowers the high lw array thresholds required for excitation—dropping from 1,060 μA to 200 μA for lwSV, and from 813 μA to 233 μA for lwST. Heshmat et al. (2020) detailed that for dendrites of 0.5 μm with average myelin thickness, only 50% of spikes cross the soma, while dendrites at 0.3 μm fail entirely. Thus, a slightly higher dendritic diameter may be more appropriate for simulating moderate degeneration. Alternative approaches, such as shortening or omitting only the peripheral nerve terminal, may also be worth exploring (Kalkman et al., 2022).

For ANFs lacking dendrites, cathodic thresholds increase further (Figure 5B), with APs typically initiated in somatic compartments. Although the pmSV array shows the smallest median increase, ST arrays always produce lower cathodic thresholds than SV arrays for complete degeneration.

Threshold variation among the 25 degenerated ANFs is greater than in healthy fibers, regardless of array location. Variable electrode-to-fiber distances remain a key factor, though correlations are less straightforward than in healthy ANFs. For completely degenerated ANFs, a positive correlation between threshold and electrode–soma distance is only observed with pmST.

It is also important to note that using alternative electrode contacts oriented toward the cochlear axis alters electrode–soma distances (see black vertical dotted lines, Figures 2E–G). Median contact–soma distance decreases from 983 to 718 μm for pmST and from 1,384 to 1,172 μm for lwST. For SV arrays, the distance increases slightly for pmSV (1,294 to 1,377 μm) and remains nearly unchanged for lwSV (1,694 μm). For completely degenerated ANFs, this change leads to slightly reduced cathodic thresholds for ST arrays, but the effect is less pronounced than in healthy ANFs.

The specificity map for thin dendrites (analogous to Figure 6) reveals more irregularities in minimum threshold values, especially for lwST. Only the most basal and some apical ANFs with thin dendrites achieve minimum cathodic thresholds in their respective target fibers. In many cases, for electrode positions between 100° and 200°, minimum thresholds are induced in ANFs around 400°, where APs are initiated in the soma and target ANFs with thin dendrites fail to propagate an AP across the soma. Thus, overall specificity is lower for thin dendrites compared to healthy ANFs. The pmST array becomes the most specific, with non-target fibers showing a median threshold increase of 228%—still lower than the 473% for healthy fibers. Specificity drops further for other array locations, with median threshold increases of 96% for pmSV, 115% for lwST, and 98% for lwSV.

This reduction in specificity is even more pronounced for completely degenerated ANFs, where almost no electrodes induce minimum thresholds in their respective target fibers. Median threshold increases are now 84% for pmST, 38% for pmSV, 81% for lwST, and 43% for lwSV. Thus, for degenerated ANFs, ST arrays are more specific than SV arrays, supporting the recommendation that pm arrays are preferable for traditional CI candidates with severe to complete hearing loss (Gibson and Boyd, 2016; Lee J. Y. et al., 2019).

### 4.3 Monophasic anodic stimulation and polarity sensitivity

Although a comprehensive discussion of monophasic anodic pulses lies beyond the scope of this study, it is important to note that anodic stimulation results in different absolute thresholds and may alter the corresponding SIS. As previously reported for lw arrays (Fellner et al., 2024), anodic stimulation produces voltage profiles along the dendrite that are opposite to those seen with cathodic pulses. Specifically, during ST stimulation, the terminal undergoes direct depolarization, initiating the AP shortly after pulse onset, whereas for SV stimulation the terminal is hyperpolarized, resulting in delayed AP initiation. The median anodic threshold for lwST is 210 μA—an average increase of 25% compared to cathodic values—while for lwSV, the median anodic threshold is 332 μA, corresponding to an average increase of 168%. Peripheral SIS shifts are uncommon and occur mainly in cases with high thresholds and brief delays in AP onset.

The scenario is somewhat more complex for pm arrays. For pmST, the terminal consistently serves as the SIS and is directly depolarized, similar to lwST, but APs are initiated slightly faster due to less pronounced hyperpolarization at adjacent nodes (with the site of maximal hyperpolarization shifted to the second or third peripheral node). The median anodic threshold for pmST is 148 μA, showing no average increase relative to cathodic stimulation.

The pmSV array exhibits high anodic thresholds, with a median of 631 μA, reflecting an average threshold increase of 146% compared to cathodic values. The SIS is usually a dendritic node, but in about a quarter of cases, it is the terminal (associated with lower thresholds), and in two instances, it is an axonal node (highest thresholds). For pmSV, the terminal may be either depolarized or hyperpolarized, and as with pmST, the site of strongest hyperpolarization is often one of the first peripheral nodes. However, high anodic currents are required because propagation across the soma is hindered by an unfavorable transmembrane voltage profile in the peripheral nodes adjacent to the pre-somatic region.

Overall, monophasic anodic stimulation with ST arrays yields lower absolute thresholds than with SV arrays. When evaluating the polarity effect (PE), defined as 𝑃𝐸 = 20·log₁₀(𝐼_cathodic / 𝐼_anodic), both SV arrays consistently show negative values. The median PE is −7.8 dB for pmSV and −8.5 dB for lwSV. For lwST, only two ANFs demonstrate small positive PE values, leading to a median PE of −1.9 dB. The median PE for pmST is slightly positive at 0.1 dB, but PE values fluctuate between positive and negative, with a decreasing trend at higher ANF angles. Thus, pmST is the only case aligning with previously reported erratic PE trends in individual geometries (Kalkman et al., 2022), although the relationship between pm and lw arrays here is reversed, with pmST displaying a higher median PE than lwST, contrary to earlier reports (Kalkman et al., 2022).

Analysis of co-stimulation of non-target ANFs with monophasic anodic pulses reveals that for the most apical electrodes of pm arrays, a frequency mismatch may occur, with minimum anodic thresholds induced in a fiber preceding the target. Nevertheless, overall specificity (quantified as the median increase in minimum thresholds) is comparable to cathodic stimulation. For anodic pulses, the pmST array is most specific (median increase of 877%), followed by lwST (527%), lwSV (362%), and pmSV (164%). This supports the conclusion that both ST arrays offer greater specificity for anodic stimulation, consistent with Kalkman et al. (2022), who observed that cross-turn stimulation thresholds are lower for monophasic cathodic than anodic stimuli. Conversely, both SV arrays are less specific for anodic than cathodic monophasic stimulation, which should be considered when selecting optimal CI pulse forms.

The SIS is almost always the terminal for target and neighboring fibers, except for pmSV, which sometimes displays peripheral or, rarely, central SISs. In all other co-stimulated fibers, APs are initiated in central nodes, the typical SIS for anodic stimulation (Potrusil et al., 2020; Heshmat et al., 2020; Kalkman et al., 2022). Kalkman et al. (2022) further noted that anodic pulses usually excite ANFs in their central axons, while cathodic stimuli tend to activate peripheral processes or regions near the cell body; as a result, cathodic thresholds are more susceptible to neural degeneration. Indeed, lower anodic thresholds for fibers with very thin (d < 0.3 μm) or completely degenerated ANFs were previously predicted in a two-dimensional homogeneous model with electrodes at various ST and SV locations (Wenger et al., 2023). However, in the present study, both cases of ANF degeneration show the opposite: thresholds increase in all but the most apical ANF when switching to anodic pulses. For severely degenerated ANFs, the average threshold increases for anodic versus cathodic stimulation is 30% for lwST and 26% for the other arrays, with PE values remaining almost always negative. In line with previous findings (Kalkman et al., 2022), neural degeneration did not consistently affect PE values, and array type (lw or pm) had less influence for degenerated fibers.

For anodic stimulation of completely degenerated ANFs, frequency mismatches are reduced compared to cathodic stimulation, with the pmST array showing the fewest outliers in minimum thresholds, which mostly occur above 450°. Median increases in minimum anodic thresholds are 48% for pmST, 20% for lwST, and 10% for both SV arrays. Thus, compared to cathodic stimulation of degenerated fibers, all arrays appear more selective and exhibit fewer frequency mismatches, though overall specificity for monophasic anodic pulses is decreased.

Additional analyses of polarity effects can be found in our recent studies, which examine lw electrodes in ST versus SV (Wenger et al., 2023; Fellner et al., 2024), as well as investigations into various pulse shapes and arrays (Heshmat et al., 2021).

### 4.4 Impact of electrode placement on threshold currents and comparison with clinical data

Our threshold current results should be compared with CI user data that exclude cognitive variables. The study by DeVries and Arenberg (2018) is relevant, reporting perceptual thresholds for 16 contacts across 13 arrays in ST and SV, using electrode-to-modiolus distance as the main factor. Electrodes closer to the modiolus (pm setting) show lower threshold currents, which aligns with research indicating that threshold decreases as fiber diameter increases (Ranck, 1975; Rattay, 1990), with human ANF axons being roughly twice the diameter of dendrites (Rattay et al., 2013). Threshold currents rise with greater electrode-to-fiber distance, so electrodes placed near ANFs yield lower thresholds. Importantly, electrode-to-modiolus distance also includes consideration of the proximity to Rosenthal’s canal, containing the soma and initial axon segments.

The threshold current is influenced by several factors, including the electrode-to-fiber distance, fiber curvature, the electrode’s proximity to the fiber ending, the density of ion channels within the sodium band, and polarity (Rattay, 1999, 2008; Werginz et al., 2014; Heshmat et al., 2021). Comprehensive evaluation of these parameters suggests that the lowest achievable threshold currents occur when a ST electrode is positioned in close proximity to the dendritic ending, specifically just beneath the organ of Corti (see increased excitability at the fiber end, Figure 12 in Fellner et al., 2024). Direct comparison between this extreme ST scenario and SV is limited by the presence of the scala media, given the necessity for identical dendrite-to-electrode distances in both configurations. However, with mid-dendritic electrode placements, it is feasible to achieve very short electrode-to-ANF distances, resulting in low thresholds. E.g., computational studies have reported values of −23.1 μA for ST and −22.8 μA for SV, whereas the optimal (“best”) pmST contact, located closest to the modiolus, requires −260 μA, more than tenfold higher. These low thresholds observed for myelinated fibers are consistent with theoretical current-distance relationships (Rattay, 1987, 1989, 2008) and corroborated by experimental findings (Ranck, 1975).

Our 3D geometry (Figures 1A, 2) indicates that the electrodes are set quite far from the cell bodies, regardless of placement. This means that, unlike our rare instances, more direct axonal stimulation is likely if the contacts are positioned closer to the modiolus. Additionally, only two human ANF 3D datasets are available (Potrusil et al., 2020), even though significant differences in cochlear structure have been reported. These differences could account for the dominance of dendritic SISs in our threshold simulations. When dendrites are lost, the SIS tends to shift toward the axons, requiring less current for pm implants compared to lw implants. At age 70, about 80% of ANF axons survive versus only 40% of dendrites (Wu et al., 2023). Many cochlear implant users may experience this 50% ratio of ANFs lacking dendrites at a younger age in certain parts of the cochlea. In these cases, a greater electrode-to-modiolus distance is likely linked to higher perceptual thresholds.

In the study by DeVries and Arenberg (2018), electrode arrays were primarily inserted into the scala tympani (ST); however, in 5 out of 13 cases, at least one of the 16 electrodes migrated into the scala vestibuli (SV). Due to the limited spatial resolution of CT imaging, some electrode positions were deemed intermediate when their scalar location could not be determined. Psychophysical tuning curves revealed that in 9 of 13 patients, threshold currents increased with greater electrode-to-modiolus distance. In about half the cases, the trends of electrode-to-modiolus distance and threshold current were similar, while in the others, threshold variations were irregular, resembling the threshold patterns observed in our Figure 3.

Notably, the lowest threshold was found at an ST electrode with the second shortest distance to the modiolus, whereas a nearby electrode with the shortest distance exhibited a threshold 30 dB higher, suggesting poor local neural innervation. In this subject, only the most apical and basal electrodes were definitively in ST, while others were intermediate or in SV, with potential neural damage associated with scalar transitions. The neural status in such cases can be further assessed using electrically evoked compound action potentials (Jahn and Arenberg, 2020).

### 4.5 Volume conductor model assumptions and ST ossification

The distribution of extracellular potential (*V_e_*) along ANFs, which drives excitation, depends on the electrical properties of the various model domains. The number and type of cochlear domains included, and thus the complexity of finite element models, varies across computational studies. Most in-silico models of the human cochlea incorporate the five domains used here (ST, SV, scala media, modiolus, bone) along with one or more additional regions such as the stria vascularis, spiral ligament, osseous spiral lamina, basilar membrane, Reissner membrane, organ of Corti, porous bone, nerve tissue, or the Rosenthal canal (Nogueira et al., 2016; Bai et al., 2020; Kalkman et al., 2014; Kalkman et al., 2022; Malherbe et al., 2013; Malherbe et al., 2015; Frijns et al., 2000; Briaire and Frijns, 2000; Ramos-de-Miguel et al., 2022; Salkim et al., 2022; Sriperumbudur et al., 2020).

While there is broad agreement on the conductivities of certain domains, bone and modiolus conductivity values show greater variability, and these domains strongly affect intrascalar potentials during stimulation (Kalkman et al., 2014). The modiolus is typically treated as a homogeneous domain, despite being a complex composite structure; Bai et al. (2020) suggest that incorporating modiolar microstructures may be necessary to fully capture ANF activity. In this model, the modiolus includes nerve tissue, membranes, and bony structures, and a low default conductivity of 0.0334 S/m was adopted based on prior models (Potrusil et al., 2020; Fellner et al., 2024). Increasing modiolus conductivity tenfold, to values used in other studies, generally raises cathodic thresholds (see Figure 7), with pm arrays showing a smaller median increase (45–50%) than lw arrays (77–95%). To further assess the impact of adding more domains and simulating a heterogeneous, possibly anisotropic modiolus, we conducted feasibility studies to be presented in future work.

The effect of bone resistivity on neural thresholds and intracochlear potentials has been previously analyzed (Malherbe et al., 2015). The authors concluded that, depending on the head model, bone resistivity values between 3,500 and 10,500 *Ω*·cm yield neural and electrical predictions consistent with measurements. They recommend a resistivity of about 10,000 Ω·cm when predicting neural excitation and about 6,500 Ω·cm for estimating electric fields within the cochlear duct. The default bone conductivity in this study corresponds to a resistivity of 6,250 Ω·cm, at the lower end of commonly assumed values. However, doubling bone conductivity to 0.032 S/m did not significantly alter cathodic thresholds (Figure 7).

To simulate varying degrees of ossification, we tested decreasing ST conductivity. Similarly, Briaire and Frijns (2000) set ST conductivity equal to bone to simulate ossification, finding reduced current densities in the ossified ST compared to their standard model. Our results also indicate that, despite surgical challenges, CI insertion into an ST with early ossification could be advantageous, resulting in lower thresholds (average decrease of 24–26%). The pmSV array generally shows lower thresholds for an ossified ST (up to 31% decrease for complete ossification), and threshold increases for lwSV arrays are modest (maximum of 10% for complete ossification). With ongoing ossification, specificity increases sharply for ST arrays; for SV arrays, specificity decreases slightly with early ossification but then rises as ossification progresses.

**Figure 9:**
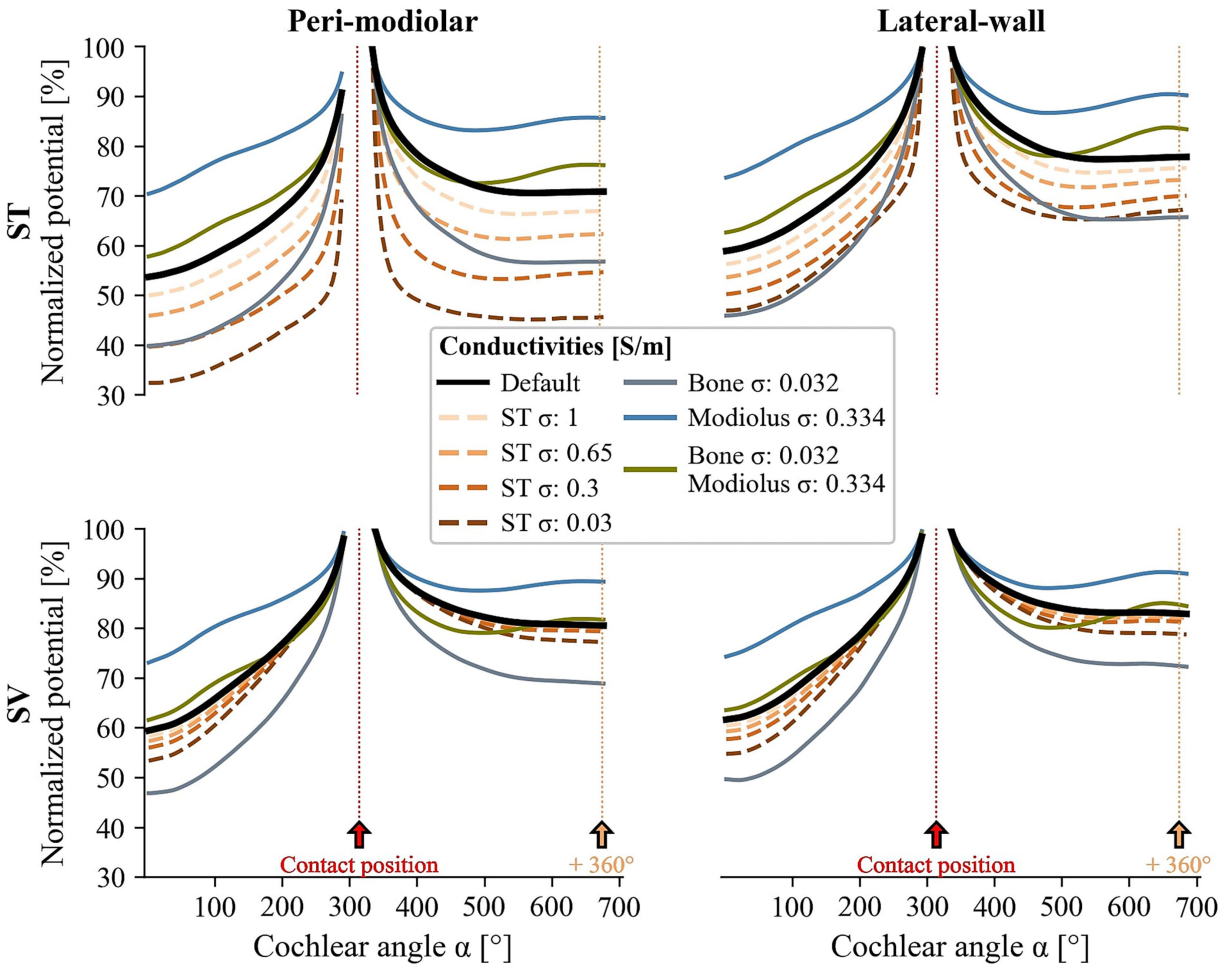
Normalized potential distributions along the electrode carrier for an active contact at an angle of 320° relative to the cochlear axis (vertical red dotted line). Results for peri-modiolar arrays are shown in the left column, and lateral-wall arrays in the right column; ST and SV array locations are presented in the upper and lower rows, respectively. Different lines correspond to varying conductivity (*σ*) assumptions for key domains in the current finite-element model; color codes are indicated in the legend. The orange dotted vertical line in each panel marks the cochlear area exactly one turn above the stimulating electrode, where a second rise in potential is evident for all arrays with increased modiolus conductivity.

The validity of our volume conductor model was previously established (Fellner et al., 2024) by comparing computed potential distributions along the lwST array with earlier measurements (Tang et al., 2011; Kalkman et al., 2014). Here, we further computed normalized potential distributions for all electrode arrays under varying modiolus, bone, and ST conductivities (Figure 9). Potentials decrease on both sides of the stimulating contact at *α* = 320°, with a more pronounced drop in the lower cochlear regions due to the cochlea’s coiled shape. The apical potential drop is most significant for the pmST array and similar for the other arrays. A tenfold increase in modiolus conductivity (blue line) results in higher potentials along the carrier, while doubling bone conductivity (gray line) reduces them. Notably, increasing both conductivities simultaneously (green line) produces potentials similar to default values. Additionally, a local maximum appears in the next turn above the stimulating electrodes at +360° (vertical orange dotted line), consistent with previous reports (Fellner et al., 2024; Tang et al., 2011; Kalkman et al., 2014) and is most influenced by modiolus conductivity.

For SV arrays, progressive ST ossification (decreasing conductivity; brown lines) only slightly reduces the induced potential compared to a patent ST. In contrast, ST arrays show a marked potential drop, especially for pmST. These findings may help explain variations in recorded potential decay (Tang et al., 2011; Kalkman et al., 2014) and could be important for identifying regions of pronounced ossification.

It is also important to note that assumptions about electrical grounding in cochlear volume conductor models have been thoroughly discussed (e.g., Nogueira et al., 2016). Placing the ground too close to the cochlea can distort intra-cochlear current flows, so, as in previous work, we set the outer bone boundary as the electric ground for monopolar stimulation, simulating a distant return electrode (Nogueira et al., 2016; Malherbe et al., 2013; Söderqvist et al., 2022). When the ground is placed at an additional contact at the basal end of the CI carrier, excitation profiles are similar (data not shown). Absolute cathodic thresholds remain largely unchanged except for the most basal ANFs near the ground, where thresholds rise. Pm arrays are more affected, with slight shifts in the SIS toward the peripheral terminal.

Finally, variations in cochlear geometry and size impact intracochlear electric field recordings (Söderqvist et al., 2022) and ANF excitation patterns (Bai et al., 2019; Kalkman et al., 2022). Future computational studies should examine the effects of SV insertion and ST ossification on simulated auditory responses in a range of cochlear models with realistic ANF populations. We hope this work encourages such investigations.

## Data Availability

The raw data supporting the conclusions of this article will be made available by the authors, without undue reservation.

## Footnotes

1
https://github.com/MPh-py/MPh
